# Occurrence rate and estimated economic burden of pulp and periapical disease treatment among Korean older adults: a national population-based retrospective study

**DOI:** 10.4178/epih.e2025035

**Published:** 2025-07-03

**Authors:** Hyeonjeong Go, Masae Kuboniwa, Youn-Hee Choi

**Affiliations:** 1 Department of Preventive Dentistry, Kyungpook National University School of Dentistry, Daegu, Korea; 2 Institute for Translational Research in Dentistry, Kyungpook National University, Daegu, Korea; 3 Department of Preventive Dentistry, Osaka University Graduate School of Dentistry, Osaka, Japan

**Keywords:** Health insurance, Cohorts, Aged, Incidence, Economics, Periapical diseases

## Abstract

**OBJECTIVES:**

Maintaining healthy dentition is essential for the overall health of older adults. Treatment of pulp and periapical disease (PPD) is crucial for preserving teeth. This study assessed the occurrence rate and economic burden of PPD treatment in an older Korean population.

**METHODS:**

Data were obtained for adults aged ≥65 years from the Korean National Health Insurance Services from 2014 to 2018. The occurrence rate was defined as the proportion of individuals who received ≥1 PPD treatment. The economic burden was estimated using both direct and indirect expenditures, calculated from a societal perspective. Regression analysis was performed to evaluate yearly trends in occurrence rate and economic burden.

**RESULTS:**

Direct costs, which included insured and non-insured medical expenses, ranged from US$13.79 million to US$10.47 million. Indirect costs, comprising transportation and time costs, ranged from US$0.89 million to US$0.80 million. Total costs declined from US$14.68 million to US$11.36 million. The occurrence rate of PPD treatment decreased significantly from 1.24% to 0.91% (p<0.05). The economic burden also demonstrated a statistically significant downward trend, with total PPD costs decreasing from 0.00099% to 0.00066% of Korea’s GDP, and from 0.63% to 0.30% of the annual total dental care benefit costs between 2014 and 2018 (p<0.05).

**CONCLUSIONS:**

The occurrence rate and estimated economic burden of PPD treatment significantly decreased among older Korean adults from 2014 to 2018. Conservative approaches to tooth preservation contribute to more effective oral health policies and help reduce the economic burden of oral disease treatments in an aging society.

## GRAPHICAL ABSTRACT

[Fig f2-epih-47-e2025035]

## Key Message

• The occurrence rate and economic burden of pulp and periapical disease (PPD) treatment among older Korean adults have shown a statistically significant decrease over the study period.

• These findings suggest that conservative dental treatment can serve as an effective oral health policy for reducing the economic burden of oral diseases in an aging society.

## INTRODUCTION

According to the World Health Organization, the global population aged 60 years and older is projected to reach approximately 2.1 billion by 2050 [1]. As of December 2024, Korea has officially entered the stage of a super-aged society, with individuals aged 65 and over constituting more than 20% of the resident registered population. Life expectancy at birth in Korea is 80.6 years for men and 86.6 years for women, both of which surpass the Organization for Economic Cooperation and Development averages of 77.7 years and 83.1 years, respectively [2]. These demographic changes have contributed to rising healthcare costs, as the prevalence of age-related diseases increases, and per capita medical expenses for those aged 65 and over continue to climb in Korea [3].

Pulp and periapical disease (PPD) treatment, which is also commonly referred to as root canal or endodontic treatment, aims to eliminate microbial biofilm infections and prevent recontamination within the complex root canal system [4,5]. This procedure is essential for preventing tooth loss [6,7] and supporting long-term tooth survival and dentition maintenance [8]. Maintaining healthy natural dentition, especially in older adults, has documented benefits for both functional and psychological health [9,10]. Each year, an estimated 15 million people worldwide undergo endodontic treatment, with about 41,000 teeth treated daily [11,12]. In Brazil and Europe, the prevalence of root-filled teeth is reported at 12% and 9.3%, respectively [13]. In Korea, the frequency of root canal–treated teeth ranges from 0.8% to 17.6%, depending on tooth type and jaw location [14]. Endodontics is among the fastest-growing fields in dentistry, underscoring a shift toward preserving teeth whenever it is clinically, technically, and economically feasible. This emphasis is particularly relevant in anticipation of an increasingly super-aged society.

Analyses of disease costs help quantify the economic burden of specific diseases or health behaviors, thereby informing policy priorities and the feasibility of prevention or treatment programs. Previous studies have shown that root canal treatment is a cost-effective option compared with tooth extraction followed by bridgework, dentures, or dental implants, as it extends tooth longevity at a lower overall cost [15]. However, relatively few studies have evaluated both the occurrence and the full spectrum of direct and indirect economic costs associated with root canal treatment, and most available research is outdated. Policymakers and those who fund dental care require updated evidence [12]. Earlier research often relied on small sample sizes or focused on short-term outcomes.

Therefore, this study aimed to assess the occurrence rate and economic burden of PPD treatment among individuals aged ≥65 years, as registered in the Korean National Health Insurance Service (KNHIS). By providing a comprehensive evaluation of both occurrence rates and the economic impact of endodontic treatment, including both direct and indirect costs, this study offers important insights into the financial and societal implications of PPD treatment. The resulting evidence-based data are intended to support informed decision-making and optimize resource allocation [16].

## MATERIALS AND METHODS

### Study population and data sources

In Korea, all citizens are enrolled in a medical insurance program, and the KNHIS provides a comprehensive health data resource. For this study, the entire Korean population aged ≥65 years registered with the KNHIS from 2014 to 2018 was surveyed. Individuals who were deceased or did not meet the relevant inclusion criteria were excluded.

We used data from the KNHIS to calculate medical expenditures. The KNHIS is a single-payer system that has provided coverage to approximately 50 million people since 2002, with 97% of individuals covered under the National Health Insurance System and the remaining 3% under Medical Aid [17,18]. The database used in this study was based on claims data from hospitals, clinics, and pharmacies, including insurance qualification data, disease codes, treatment codes, prescription records, and medical charges from 2014 to 2018. Disease codes in the KNHIS were derived from the Korean Standard Classification of Disease, 7th revision (KCD-7), which is adapted from the International Classification of Diseases, 10th revision (ICD-10). The study data were customized to extract only the necessary information from the KNHIS.

We utilized data from the Korea Health Panel Survey (KHPS) to estimate indirect costs. This annual, nationwide panel survey has been jointly conducted by the National Institute for Health and Social Research and the KNHIS since 2008, providing a representative sample of the population. We obtained the employment rate for adults aged ≥65 years from Statistics Korea [19], and the minimum hourly wage from the Ministry of Employment and Labor [20].

### The operational definition of pulp and periapical disease treatment

PPD was identified using KCD-7 disease codes. We defined PPD as the presence of the following codes: K040 (pulpitis), K041 (necrosis of pulp), K044 (acute apical periodontitis of pulpal origin), K045 (chronic apical periodontitis), K046 (periapical abscess with sinus), and K047 (periapical abscess without sinus). Other codes under K04, such as K042, K043, and K048, which pertain to dental pulp degeneration, abnormal formation, and radicular cysts, were excluded as they do not correspond to PPD.

Defining PPD based solely on diagnosis codes, rather than including treatment codes, allows for the identification of both treated and untreated individuals. This approach is particularly useful for estimating not only the total medical expenditures incurred during the diagnostic process but also the overall prevalence of the disease. By not limiting the definition to treatment codes, all healthcare expenditures associated with the disease, as captured by the diagnosis code, can be comprehensively included—regardless of whether actual treatment was administered. Additionally, if both diagnosis and treatment codes are used in the operational definition, some diagnosis-related costs may be excluded from total expenditure calculations. To prevent such limitations and ensure a more complete assessment of disease burden, PPD was operationally defined using diagnosis codes alone.

### Direct costs

We used the KNHIS-customized database to calculate the medical expenditures for PPD treatment in adults aged ≥65 years with a history of PPD diagnosis from January 1, 2014, to December 31, 2018. The total annual medical care benefits for individuals with relevant disease codes were collected. This included both national and out-of-pocket costs, and refers to the amount determined by the Health Insurance Review and Assessment Service after reviewing the total charges from medical institutions, excluding pharmacy costs. To estimate non-insured medical expenditures for PPD treatment, the patient responsibility rate and non-covered patient responsibility rate were taken from the Health Insurance Patient Medical Expenses Survey report [21] in the KNHIS and used to calculate non-insured medical costs.

### Indirect costs

Transportation costs included all expenses incurred by patients when traveling to healthcare facilities for treatment. Time costs represented the opportunity cost associated with time lost during these visits. Indirect costs, such as transportation and time costs related to dental hospital visits from 2014 to 2018, were obtained from the KHPS.

According to dental health insurance policies, root canal irrigation is covered by insurance for up to 5 sessions. Since not all patients require the maximum number of sessions, we conservatively estimated 2 visits based on typical clinical practice in Korea. Previous studies have defined multiple-visit endodontic treatment as involving 2 or more sessions, which supports our use of 2 visits as a reference point [22]. Therefore, we multiplied the number of PPD treatment visits by 2 when calculating time and transportation costs.

We calculated the average round-trip transportation cost per person for visits to the dental clinic, and this figure was doubled to account for 2 visits for PPD treatment. The average time required for each PPD treatment—including cavity preparation, pulp extirpation, working length determination, and root canal shaping and filling—was derived from prior studies [23,24]. The average time cost per person was calculated as the minimum time needed for 2 dental visits, including transportation time. This time was then multiplied by the employment rate for those aged ≥65 years and by either the average hourly wage or the minimum wage. The average transportation and time cost per person was finally multiplied by the total number of individuals identified in the KNHIS to estimate the total transportation and time costs for the entire population of older adults receiving PPD treatment.

### Statistical analysis

We conducted a cost analysis from a societal perspective, following the healthcare cost calculation methods recommended by the National Evidence-based Healthcare Collaborating Agency (NECA) [25,26]. The economic cost of PPD treatment for all older adults in Korea was calculated by summing the annual direct and indirect costs and applying a 5% discount rate to present all costs at their current value.

The occurrence rate of PPD was calculated as the number of new individuals who experienced PPD at least once per year, divided by the total population at risk and multiplied by 100. The total population at risk was estimated by subtracting the number of edentulous individuals from the total population aged ≥65 years. The proportion of edentulous individuals was derived from the study by Yu et al. [27], which reported weighted estimates of edentulism in the Korean population using data from the Korea National Health and Nutrition Examination Survey between 2007 and 2018. We specifically used the annual weighted proportions of edentulous individuals for the years 2014 to 2018, as reported in the study. As shown in Table 1, to estimate the number of edentulous individuals, these annual proportions were multiplied by the mid-year population figures (mid-year estimates) for each year from 2014 to 2018, as reported by Statistics Korea. The estimated number of edentulous individuals was then subtracted from the mid-year population aged ≥65 years to obtain the population at risk—those who still retained teeth and were therefore eligible to receive PPD treatment.

Yearly gross domestic product (GDP) figures were obtained from the International Financial Statistics of the Bank of Korea, while the annual total costs of dental care benefits (ATCD) were sourced from Healthcare Cost Major Statistics, provided by the Korean Health Insurance Review and Assessment Service. The average annual percent change (AAPC) was calculated to represent the trends observed between 2014 and 2018. The AAPC was determined by averaging the yearly percentage changes in rates from the baseline to the final year, providing a summary measure of the trend over the study period. Descriptive statistics were used to analyze the number of cases (n), mean costs, and total costs. All costs were converted into United States dollars (US$) using the average annual exchange rate provided by International Financial Statistics. To evaluate trends in the occurrence rate of PPD and its associated economic burden, linear regression analysis was performed as a statistical trend test. In this analysis, the year was treated as an independent variable, and outcome measures—including the PPD occurrence rate and economic burden—were used as dependent variables to assess whether statistically significant trends existed over time.

We utilized SAS version 9.4 (SAS Institute Inc., Cary, NC, USA) for all statistical analyses with a statistical significance level of p-value <0.05.

### Ethics statement

This study was conducted in accordance with the Declaration of Helsinki, and the study protocol was approved by the Institutional Review Board/Ethics Committee of Kyungpook National University (KNU-2021-0489). Informed consent was waived because this was a retrospective study utilizing deidentified administrative data.

## RESULTS

Table 1 presents the annual number of individuals receiving PPD treatment, the total population at risk, mean cost, standard deviation, insured medical costs, and estimated non-insured medical costs for PPD among adults aged ≥65 years in Korea. Over the study period, the average AAPC in the total population at risk among older Korean adults increased by 3%, while the AAPC for older adults receiving PPD treatment declined by 3% from 2014 to 2018. The total insured medical costs for PPD treatment, as reported by the KNHIS, decreased from US$5.43 million to US$4.56 million. The estimated non-insured expenditures for PPD treatment, including prostheses such as crowns, declined from US$8.36 million to US$5.92 million during the same period.

As shown in Table 2, the average one-way transportation cost per person, estimated from KHPS data, ranged from US$1.68 to US$1.58 between 2014 and 2018. The average round-trip transportation cost per person for 2 dental clinic visits decreased from US$6.71 in 2014 to US$6.33 in 2018. According to Table 3, the average one-way time cost (in minutes) per person for a dental clinic visit, based on KHPS data, increased slightly from US$18.63 to US$19.16 between 2014 and 2018. The average round-trip time cost (in hours) per person for 2 dental clinic visits changed minimally, from US$2.74 in 2014 to US$2.78 in 2018. When calculated using the average hourly wage and adjusted for time and employment rate, the total time costs ranged from US$22.45 in 2014 to US$18.48 in 2018. When calculated based on the minimum wage, the total time costs increased from US$5.99 in 2014 to US$8.33 in 2018.

**Figure 1.**
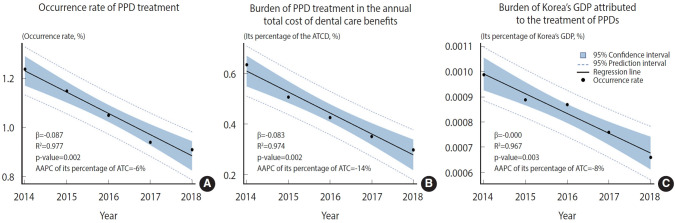
Trend of occurrence rate and economic burden of pulp and periapical disease (PPD) treatment among older adults in Korea. (A) Is the occurrence rate of PPD treatment. It was calculated by dividing the number of new individuals who experienced PPD at least once per year by the total population at risk, then multiplying by 100. (B) Is the proportion of annual dental care expenditure accounted for by PPD treatment. The annual total cost of dental care does not include non-covered expenses. (C) Is the proportion of PPD treatment costs in relation to the gross domestic product (GDP). AAPC, average annual percent change; ATCD, annual total cost of dental care benefits. p<0.05 for the regression analysis for the trend test.

Table 4 summarizes the total costs, which include the sum of PPD treatment expenditures, transportation expenses, and time costs. The total costs ranged from US$14.68 million in 2014 to US$11.36 million in 2018, with an AAPC of -5%. After applying the 5% discount rate, the total costs were US$9.01 million in 2014 and US$8.48 million in 2018, with an AAPC of -1%. As shown in Figure 1A, the occurrence rate of PPD treatment significantly declined from 1.24% to 0.91%, corresponding to an AAPC of -6% (β=-0.085; p=0.002; R²=0.977). The burden of total PPD costs as a proportion of ATCD in Korea also significantly decreased from 0.6344% to 0.2971%, reflecting an AAPC of -14% (β=-0.083; p=0.002; R²=0.974), as shown in Figure 1B. Figure 1C demonstrates that this expenditure accounted for 0.00099% to 0.00066% of the national GDP, with an AAPC of -8% (β=-0.083; p=0.002; R²=0.974).

## DISCUSSION

We estimated the trend in total costs associated with PPD treatment among individuals aged ≥65 years from 2014 to 2018 using data from the KNHIS and KHPS. Over this period, the occurrence rate of PPD treatment among older Korean adults declined from 1.24% to 0.91%. The estimated total costs for PPD treatment fell from US$14.68 million to US$11.36 million, while the discounted total cost decreased by approximately 1%, from US$9.01 million to US$8.48 million. The economic burden of PPD treatment also decreased, representing 0.6344% to 0.2971% of the annual total cost of dental care benefits, and 0.00099% to 0.00066% of the Korean GDP.

A previous systematic review reported that the global prevalence of adults with at least 1 endodontic treatment was 55.7% [13]. However, the frequency of root canal treatment varies widely among countries, with the proportion of root-filled teeth ranging from 0.7% [28] to 87% [29]. Another systematic review estimated the incidence of pulp necrosis and periapical lesions to be between 3.63% and 5.02% [30]. According to a global trend analysis based on data from the Global Burden of Disease Study, the age-standardized prevalence of dental caries decreased by 3.6% from 1990 to 2019 [31]. In Korea, a KNHIS-based analysis found that the incidence of advanced dental caries requiring endodontic treatment, such as root canal therapy, ranged from 4.5% to 6.2% [32]. Similarly, data from the Korea National Health and Nutrition Examination Survey showed that the prevalence of permanent tooth caries decreased from 31.4% in 2014 to 29.1% in 2018, indicating a 2.3% reduction [33]. These national and global trends are consistent with our findings. In the present study, the occurrence rate of PPD treatment in older adults ranged from 1.24% to 0.91%, demonstrating a decreasing trend from 2014 to 2018.

To estimate indirect costs, we included transportation expenses and time costs in our analysis. Time costs, typically proportional to travel distance and wage [34], represent a significant component of the overall indirect burden of PPD treatment. Older adults often experience barriers to healthcare access related to time, distance, and transportation [35-37]. The requirement for multiple visits during PPD treatment increases both transportation and time costs, imposing a substantial burden on patients. Prior research has shown that older individuals and rural residents are less likely to seek health screenings or medical care for mild symptoms, contributing to increased disease severity and poorer outcomes [38]. If dental decay remains untreated, it can progress to infection and inflammation, necessitating endodontic intervention and resulting in greater expense and time commitment. For these reasons, the indirect costs associated with PPD care are likely significant for older adults. Our estimates provide a conservative baseline, and the true burden of indirect costs in this population is likely higher.

In our study, the total cost of PPD treatment decreased from US$14.68 million to US$11.36 million between 2014 and 2018, with an AAPC of –5%. The economic burden of PPD treatment relative to the annual total cost of dental care benefits declined by 14%, and its proportion relative to Korea’s GDP decreased by 8%. In contrast, a prior Korean study [39] found that out-of-pocket expenditures for implant treatments among older adults rose significantly following the expansion of National Health Insurance coverage, while spending on conservative treatments decreased. Other Korean research has reported consistent increases in implant costs during the same period [40]. Thus, the observed reduction in the occurrence rate and economic burden of PPD treatment may have resulted from advances in implant procedures, greater awareness of their advantages, and expanded insurance coverage for implants. In some cases, implants may have been chosen instead of endodontic treatment for teeth that could not be preserved, or for cases involving challenging root canal therapy. Improvements in oral hygiene practices may also have contributed to the reduced need for PPD treatment. For example, the introduction of insurance-covered dental scaling in July 2013 has been shown to promote positive oral health behaviors in Korea [41]. Enhanced oral hygiene management improves oral health status and may lower the incidence of PPD treatment. These combined factors may explain the relative declines in both the economic burden and occurrence rate of PPD treatment observed in our study. Nevertheless, older adults still demonstrate a markedly higher prevalence of PPD compared to younger populations, and treatment outcomes are generally more favorable among older adults [42,43]. Therefore, PPD treatment may continue to offer significant value for older adults in Korea.

The AAPC in the total discounted costs of PPD treatment from 2014 to 2018 decreased by approximately 1%, although this change was not statistically significant. While the occurrence rate of PPD declined and both the total costs and economic burden showed downward trends, the total costs after applying the discount rate did not display a consistent or significant trend. This is attributed to the application of a 5% discount rate, which was used to adjust historical costs to their present value. Although the actual trend in total costs is decreasing, the trend may appear irregular when evaluated in present value terms (as of 2024). This inconsistency arises from the assumption of a 5% annual inflation rate in accordance with NECA cost analysis guidelines [25,26]. The non-linear application of the discount rate and resulting fluctuations in currency value contribute to this variability. Specifically, the compounded effect of applying the 5% discount rate over multiple years causes present value calculations to fluctuate non-linearly. This approach was intended to express the monetary value of each year in present-day currency. However, because the discount rate is an assumed value and can distort temporal comparisons, undiscounted total costs were used in this study to evaluate trends more accurately and reflect the actual changes over time.

This study uses a population-based cohort dataset from the KNHIS, which allowed for calculation of both occurrence rates and total medical costs among all older adults in Korea. The database is extensive, comprehensive, and stable, as it is derived from National Health Insurance records managed by government and public institutions. Additionally, we evaluated indirect costs incurred by older individuals accessing dental healthcare services using the KHPS, a nationally representative dataset of individuals and households in Korea. To our knowledge, this study represents a significant strength as the first cost-of-illness study to quantify the magnitude of the economic burden of PPD treatment from a societal perspective using large-scale data. Adopting a societal perspective is crucial when estimating healthcare costs, as it accounts for broader impacts, including patient well-being, quality of life, and indirect costs beyond direct medical expenses. This approach offers a more comprehensive understanding of the economic implications of healthcare interventions [44]. Consequently, these findings may provide valuable insights for policymakers developing more effective health policies. Moreover, our results support improvements in oral health-related quality of life for older adults and enable timely responses to the challenges posed by an aging society.

There are several limitations to this study. First, we estimated the medical costs of PPD treatment using diagnosis codes alone, without incorporating treatment codes. Relying solely on treatment codes may underestimate total costs due to the potential omission of relevant procedures, whereas diagnosis codes capture the full range of associated costs, providing a more comprehensive estimate of total medical expenditures for PPD treatment. Although this approach may limit the ability to assess detailed clinical context and disease severity, it allows for robust epidemiological evaluation at the population level. The intent of this analysis is to support effective policy planning, resource allocation, and preventive strategies. Because the data were derived from the National Health Insurance Service, which covers the entire Korean older population, the findings are both representative and meaningful for informing public oral health decision-making. Second, this study focused on individuals rather than teeth, which may have led to underestimation of the occurrence rate and burden of PPD treatment. While a tooth-level analysis would provide a more precise estimate of the occurrence rate and burden, an individual-level approach more effectively captures the overall impact of PPD within a population from a public health perspective. Individuals with multiple affected teeth may disproportionately influence the data, potentially resulting in overestimation of the burden compared to those with healthier oral conditions. Given our objective of generating epidemiological evidence to inform public health policy decisions in oral health, we determined that estimating the burden at the individual level would be more appropriate for the purposes of this study. However, future analyses at the tooth level may yield more precise estimates of disease occurrence and burden, highlighting the need for further research to provide more detailed insights. Third, cases of PPD not captured in the insurance claims data—including individuals who did not receive a diagnosis or treatment—may have been excluded from estimates of occurrence rates and the burden of healthcare costs. As a result, the actual burden of PPD in the population is likely higher than our estimates suggest. Fourth, we calculated the economic burden with respect to GDP and the annual total costs of dental care benefits for the entire population, making it difficult to precisely determine the cost burden specifically for older adults. Nevertheless, this study evaluates the societal burden of PPD, considering individuals aged ≥65 years as a non-economically active group requiring caregiving.

This study indicates that the occurrence rate and economic burden of PPD treatment among older adults have steadily decreased. The results highlight the significant impact of PPD treatment on total societal costs and may help inform decisions regarding the implementation of oral health policies and programs for older adults. To prepare for a healthy aging society, it is essential to continuously monitor the occurrence rate and economic burden of PPD treatment as part of efforts to maintain good oral health in the older population.

## Figures and Tables

**Figure f2-epih-47-e2025035:**
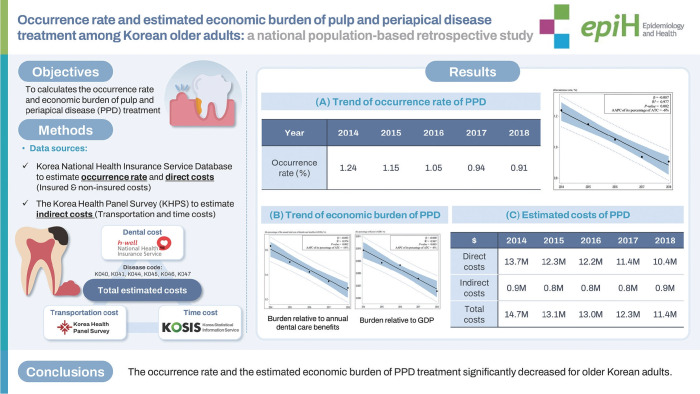


**Table 1.** Estimated medical costs of PPD treatment for individuals aged 65 and older

Year	No. of individuals with PPD	Total population	Edentulous population	Total population at risk^1^	Insured medical costs (US$) for PPD covered by the KNHIS		Non-insured medical costs (US$) for PPD^2^	
					Mean±SD^3^	Total^3^	Non-insured cost burden rate relative to insured (%)	Estimated costs^3^
2014	70,408	6,277,126	583,773	5,693,353	77.06±74.19	5,425,331.70	60.65	8,364,799.85
2015	68,051	6,541,168	644,305	5,896,863	73.10±68.70	4,974,283.58	59.40	7,277,646.42
2016	64,145	6,757,083	656,788	6,100,295	72.86±70.29	4,673,449.83	61.80	7,560,712.03
2017	60,143	7,066,060	686,821	6,379,239	76.65±73.92	4,610,002.06	59.70	6,829,209.01
2018	60,757	7,366,085	715,983	6,650,102	74.99±71.80	4,556,073.68	56.50	5,917,658.92

PPD, pulp and periapical disease; KNHIS, Korean National Health Insurance Service; SD, standard deviation.

1 Total population at risk: the total population aged ≥65 years (as of mid-year population from Statistics Korea)–Edentulous population aged ≥65 years (who were calculated using the edentulous prevalence for those aged ≥65 years; as presented by Yu et al. [27])

2 The patient responsibility rate and non-covered patient responsibility rate were calculated from the Health Insurance Patient Medical Expenses Survey report by the KNHIS to estimate the cost of non-insured medical expenditures for PPD.

3 Costs were converted into United States dollars (US$) using the average exchange rate for the year of pulp and periapical diseases treatment from International Financial Statistics; The Bank of Korea, IMF International Financial Statistics 2022.7.

**Table 2.** Estimated transportation costs^1^ regarding dental clinic utilization per person

Variables	2014	2015	2016	2017	2018
Total (n)	4,731	4,703	4,507	4,234	4,678
One-way cost	1.68	1.49	1.54	1.71	1.58
Round-trip cost	3.36	2.98	3.09	3.42	3.16
Round-trip cost×2 times	6.71	5.97	6.18	6.84	6.33

1 The costs were converted into United States dollars (US$) using the average exchange rate for the year from International Financial Statistics; The Bank of Korea, IMF “International Financial Statistics” 2022.7.

Data sources: Korean Health Panel Survey 2014-2018, dental clinics, including dental hospitals, dental clinics, and dental offices.

**Table 3.** Estimated time costs for pulp and periapical disease (PPD) treatment^1^ per person

Variables	2014	2015	2016	2017	2018
Total (n)	4,731	4,703	4,507	4,234	4,678
One-way time (min)	18.63	19.03	19.09	19.05	19.16
Round-trip time (min)×2 times	74.52	76.12	76.36	76.2	76.64
Total time (hr)^2^	2.74	2.77	2.77	2.77	2.78
Total time costs 1^3^	22.45	18.69	17.47	16.83	18.48
Total time costs 2^4^	5.99	6.00	6.30	6.82	8.33

Unit: United States dollars (US$).

1 The treatment time for PPD is, on average, 90.0 minutes; from Wong et al. [23] and Im et al. [24].

2 Total time=PPD treatment time (90 minutes)+round-trip travel time.

3 Time×Employment rate×Average hourly wage per person.

4 Time×Employment rate×Hourly minimum wage per person.

**Table 4.** Estimated economic costs for pulp and periapical disease treatment among older adults

Cost items	Year				
	2014	2015	2016	2017	2018
Insured medical expenses	5,425,331.70	4,974,283.58	4,673,449.83	4,610,002.06	4,556,073.68
Non-insured medical expenses	8,364,799.85	7,277,646.42	7,560,712.03	6,829,209.01	5,917,658.92
Transportation cost	472,437.68	406,264.47	396,416.10	411,378.12	384,591.81
Time cost	421,743.92	408,306.00	404,113.50	410,175.26	506,105.81
Total cost	14,684,313.15	13,066,500.47	13,034,691.46	12,260,764.45	11,364,430.22
Total cost after discount^1^	9,014,894.46	8,422,782.71	8,822,392.25	8,713,496.39	8,480,312.80

United States dollars (US$), the average annual percent change between 2014 and 2018.

1 The costs were assessed at their present value (2024) through the application of a 5% discount rate.
